# Implicit and explicit ethnic biases in multicultural primary care: the case of trainee general practitioners

**DOI:** 10.1186/s12875-022-01698-8

**Published:** 2022-04-21

**Authors:** Camille Duveau, Stéphanie Demoulin, Marie Dauvrin, Brice Lepièce, Vincent Lorant

**Affiliations:** 1 Institute of Health and Society, UCLouvain, Brussels, Belgium; 2 Faculty of Psychology and Educational Sciences, UCLouvain, Louvain-la-Neuve, Belgium; 3 Belgian Health Care Knowledge Centre, KCE, Brussels, Belgium

**Keywords:** General Practitioner, Discrimination, Ingroup relationship, Migrant health, Racial bias, Cultural competence, Implicit association test

## Abstract

**Background:**

General Practitioners (GPs) are the first point of contact for people from ethnic and migrant groups who have health problems. Discrimination can occur in this health care sector. Few studies, however, have investigated implicit and explicit biases in general practice against ethnic and migrant groups. This study, therefore, investigated the extent of implicit ethnic biases and willingness to adapt care to migrant patients among trainee GPs, and the factors involved therein, in order to measure explicit bias and explore a dimension of cultural competence.

**Methods:**

In 2021, data were collected from 207 trainee GPs in the French-speaking part of Belgium. The respondents passed an Implicit Association Test (IAT), a validated tool used to measure implicit biases against ethnic groups. An explicit attitude of willingness to adapt care to diversity, one of the dimensions of cultural competence, was measured using the Hudelson scale.

**Results:**

The overwhelming majority of trainee GPs (82.6%, 95% CI: 0.77 – 0.88) had implicit preferences for their ingroup to the detriment of ethnic and migrant groups. Overall, the majority of respondents considered it the responsibility of GPs to adapt their attitudes and practices to migrants’ needs. More than 50% of trainee GPs, however, considered it the responsibility of migrant patients to adapt to the values and habits of the host country.

**Conclusions:**

This study found that the trainee GPs had high to very high levels of implicit ethnic bias and that they were not always willing to adapt care to the values of migrants. We therefore recommend that they are made aware of this bias and we recommend using the IAT and Hudelson scales as educational tools to address ethnic biases in primary care.

**Supplementary Information:**

The online version contains supplementary material available at 10.1186/s12875-022-01698-8.

## Introduction

As aspiring health care professionals, trainee General Practitioners (GPs) care for a diverse and multicultural population in their practice [1] and are usually the first point of contact for ethnic and migrant patients within the health care system. They are, therefore, key to the equity of the health care system. Nevertheless, even GPs who are well-intentioned may be vulnerable, like anyone else, to implicit biases and may lack willingness to adapt care to migrants, a willingness that is a prerequisite of cultural competence [2]. This could lead GPs to discriminate unintentionally against their ethnic and migrant patients [3, 4] especially if they are under time pressure, lack solid knowledge/information needed to make a decision, or are affected by cognitive overload or fatigue [5], or due to inappropriate intercultural contact [6] less concordant physician–patient ethnicity, which is associated with less favourable patient ratings care [7], stereotyping, prejudice, political attitudes [8], or organizational and institutional factors [9]. Implicit ethnic biases among health care professionals and cultural competence are important factors that contribute to health care disparities [10, 11].

In social psychology, a “bias” is defined as a prejudicial attitude towards a group (hereafter, the exogroup) and its members, of which the holder may or may not be aware. Implicit bias is often automatically activated and can lead to impaired judgement and to negative evaluation of the exogroup [12]. Implicit ethnic bias has been shown to play a role in differential recommendations by physicians for managing disease [10], disparities in empathy [13] and differential drug prescriptions, interaction with patients, and treatment decisions [10].

To address these differences in care for ethnic/migrant groups, the report *Unequal Treatment* by the Institute of Medicine recommended exploring healthcare providers’ conscious (explicit bias) and unconscious perceptions [14]. This report also recommended developing and enhancing cultural competence training for health care professionals. Over the past decade, many cultural competence programmes have been initiated in health care systems to improve the relationships between health care professionals and patients with diverse cultural backgrounds, and to enable them to work and interact more effectively with those patients [11]. These programmes, however, are based on the assumption that health professionals are motivated and accept responsibility for adapting to ethnic diversity [15], an assumption that may need to be verified. This study aims to add to the literature by measuring implicit and explicit biases against ethnic/migrant groups.

A large body of research has investigated unconscious biases and levels of cultural competence among health care professionals. This research found significant evidence of implicit bias among care professionals against African Americans, ranging from slight bias to high levels of bias, but found low levels of explicit bias against this ethnic group. The research also found a weak association between self-perceived cultural competence and levels of implicit bias [10, 16]. Little is known, however, about these issues within the European context and a recent study comparing clinicians’ racial biases in The United States and France concluded that health providers’ biases differed between cultures and countries [17].

So far, most health care studies have been carried out in the United States and have compared African American patients with White patients. There is a lack of data on unintentional ethnic and racial discrimination among general practitioners in Europe and too little attention has been paid to the North African population. This applies particularly to people of Moroccan descent, who constitute the largest and fastest-growing minority group in countries in the European Union (EU), including Belgium, where the Moroccan population doubled between 1991 and 2014.^1^ This ethnic group is also disproportionately at risk of poor health outcomes, such as more depressive symptoms [18] and higher mortality caused by diabetes and infectious diseases [19].

To the best of our knowledge, few studies have investigated the extent of bias among general practitioners against racial/ethnic groups in relation to GPs’ willingness to adapt to diversity. A recent study highlighted the need to increase recognition and awareness of ethnic disparities in health and health care among health care professionals [20]. This paper aims to expand this area of research by investigating (a) the level of implicit ethnic bias among trainee GPs and (b) whether this level of bias varies as a function of a GP’s willingness to adapt care to migrants or as a function of openness to cultural diversity which, in this paper, includes intercultural contact, political opinions, and workload (time pressure).

## Methods

### Design & participants

This prospective quantitative study used an observational design. The design measures implicit ethnic biases and the specific explicit attitude of willingness to adapt to diversity, a prerequisite of cultural competence. To encourage trainee general practitioners to respond to the online survey, we offered to present the overall results to them anonymously and give them an opportunity to comment on and discuss the results in small groups.

Between February and March 2021, we contacted 220 French-speaking Belgian GPs, who were in the second year of their three-year internship by email in one of the important French-speaking universities in Belgium. Of those trainees, 207 completed the online survey (a participation rate of 94%) (Male = 68 (33.0%); Female = 139 (67.0%); Mean age = 26.7 years (± 2.1); See Table 1).

**Table 1:** Demographic characteristics, implicit bias, and cultural competence score of participants (*n* = 207)

Characteristics	% or mean (std)
Age (years)	26.7 (2.1)
Sex female	67.0
*Type of practice*	
Solo	27.7
Group	69.4
Mixed	2.9
*Ethnicity*	
White	78.5
Arab	8.3
African	3.9
Mixed or other	9.3
*Religion*	
Don’t want to reply	4.4
Catholic	39.1
Muslim	10.1
Atheist	31.9
Other	14.5
*Political opinion*	
Don’t want to reply	10.6
Right-wing liberal	29.5
Centre	31.9
Left-wing (e.g. socialist)	28.0
*Practice area*	
Urban	7.8
Suburban	33.1
Rural	25.3
Missing values	33.8
Frequency of contact with North Africans (/4)	2.5 (1.2)
Workload (no. of patients/day)	10.2 (5.6)
Proportion of patients of foreign origin (/100)	33.0 (30.4)
Implicit Association Test score (-2, 2)	0.53 (0.44)
Hudelson score (5, 35)	18.9 (5.1)

### Measures

#### Implicit association test

The Implicit Association Test (IAT) is a validated tool in that is used, in social psychology, to assess implicit biases, which can be associated with a wide range of behaviours and attitudes [21]. As bias is often activated by situational cues such as a first name [3], respondents took an IAT with French-language and North African first names.^2^ The IAT was designed to measure implicit ethnic bias. The IAT with North African first names uses reaction times to assess the strength of automatic associations between target pairs, each consisting of an ethnicity (e.g. French-language or Belgo-Moroccan first names), and a category (e.g. positive or negative). This test provides a D-score ranging from -2 (respondent has implicit associations of positive words with North African first names) to + 2 (respondent has implicit associations of positive words with French-language first names). We used standard cut-offs to classify the D-scores according to the strength of the implicit associations: “neutral” (< 0.15), “slight” (< 0.35), “moderate” (< 0.65), and “strong” (≥ 0.65), and the reverse for scores with a negative value [22].

#### Hudelson scale^3^

The trainee GPs completed the validated Hudelson scale, which assesses one dimension of cultural competence: the perception of relative responsibility for adapting care to migrant patients [2]. This was assessed using descriptions of five potential situations: (1) when migrants' values and habits differ from those of the host country, (2) when the patient does not speak the language of the host country, (3) when the patient expresses the wish to be treated by a male/female health professional, (4) when the patient cannot read the language of the host country, and (5) when the patient's health beliefs contradict the knowledge of the health professionals. The trainee GPs had to state their opinion about whose responsibility it is to adapt in those situations by ticking a score between 1 (it is the professional’s responsibility) and 7 (it is the migrant’s responsibility), with 4 meaning that the responsibility is shared between the patient and the professional.

Regarding openness to cultural diversity, we expected left-wing political opinions, the proportion of migrants among the patients GP cared for, more social interactions with people of North-African origin and a lighter workload to be associated with less unintentional discrimination [23]. To compute the frequency of intercultural contact, we created a score composed of four variables: (1) presence of North-African people in their neighbourhood, (2) daily interaction with North-African people, (3) having North-African friend(s), and (4) having North-African colleague(s). We obtained a score out of 4 points (where 0 means that the respondent has no contact with North Africans and 4 means that the respondent frequently interacts with North Africans). Control variables were added to the models, such as age, sex, ethnicity, and type of practice (e.g. solo, group, or mixed), to avoid potential selection bias.

#### Reactions and feedback sharing

The trainee GPs were presented with feedback on the aggregate results and then asked to comment on the results. We collected 204 trainee GPs’ anonymized open-ended reactions to their IAT and Hudelson scale scores. We then divided them into small groups of 4–5 to discuss the experience and explore possible solutions.

### Statistical analysis

#### Quantitative analysis

First, we computed descriptive statistics of variables. We computed correlations between the level of implicit bias and the Hudelson score to check whether they overlapped. Then, linear regression was performed to relate the two outcomes (implicit and explicit attitudes) with the following variables: political opinions, practice area, frequency of intercultural contact, workload, and proportion of patients of foreign origin, controlling for variables such as sex, type of practice, and ethnicity. We ran one model for each of the following explanatory variables: political opinion, frequency of intercultural contact with North Africans, frequency of patients of foreign origin in consultations. Statistical analysis was performed using SAS 9.4.

#### Reactions and feedback sharing

With the aid of NVIVO, we grouped similar quotes from the written reactions into main themes in order to explore the general reactions to the results and the possible solutions that trainee GPs highlighted within the small groups.

## Results

### Participants

Two hundred and seven General Practitioners in the second year of a three-year specialisation in general medicine responded to the online questionnaire. Of those respondents, two hundred and four took part in the discussion about the presentation of their results from the IAT and the Hudelson scale. Most of the participants were of Belgian nationality (89.0%). Participants self-reported as White (78.5%), Arab (8.3%), or of mixed/other ethnicity (9.3%). A third of the respondents self-reported as Catholic (39.1%). Nearly 70% of the respondents were not very religious or not religious at all. In terms of political opinion, the majority selected Centre (31.9%) and 29.5% of the sample identified as right-wing.

Most worked in suburban areas (33.1%). A quarter of the respondents worked in rural areas. A minority (7.8%) worked in the seven largest cities in the Wallonia-Brussels Federation (Brussels, Liège, Verviers, Namur, Charleroi, Mons, and Tournai). The area was computed based on their medical practice’s postcode.

The average score for frequency of contact with North Africans was 2.5/4 (± 1.2), meaning that the respondent sometimes interacts with North African friends, colleagues, or neighbours. In their practice, they visit 10.2 patients per day on average, 33% (± 30.4) of whom are foreign patients.

### Implicit association test

**Fig. 1:**
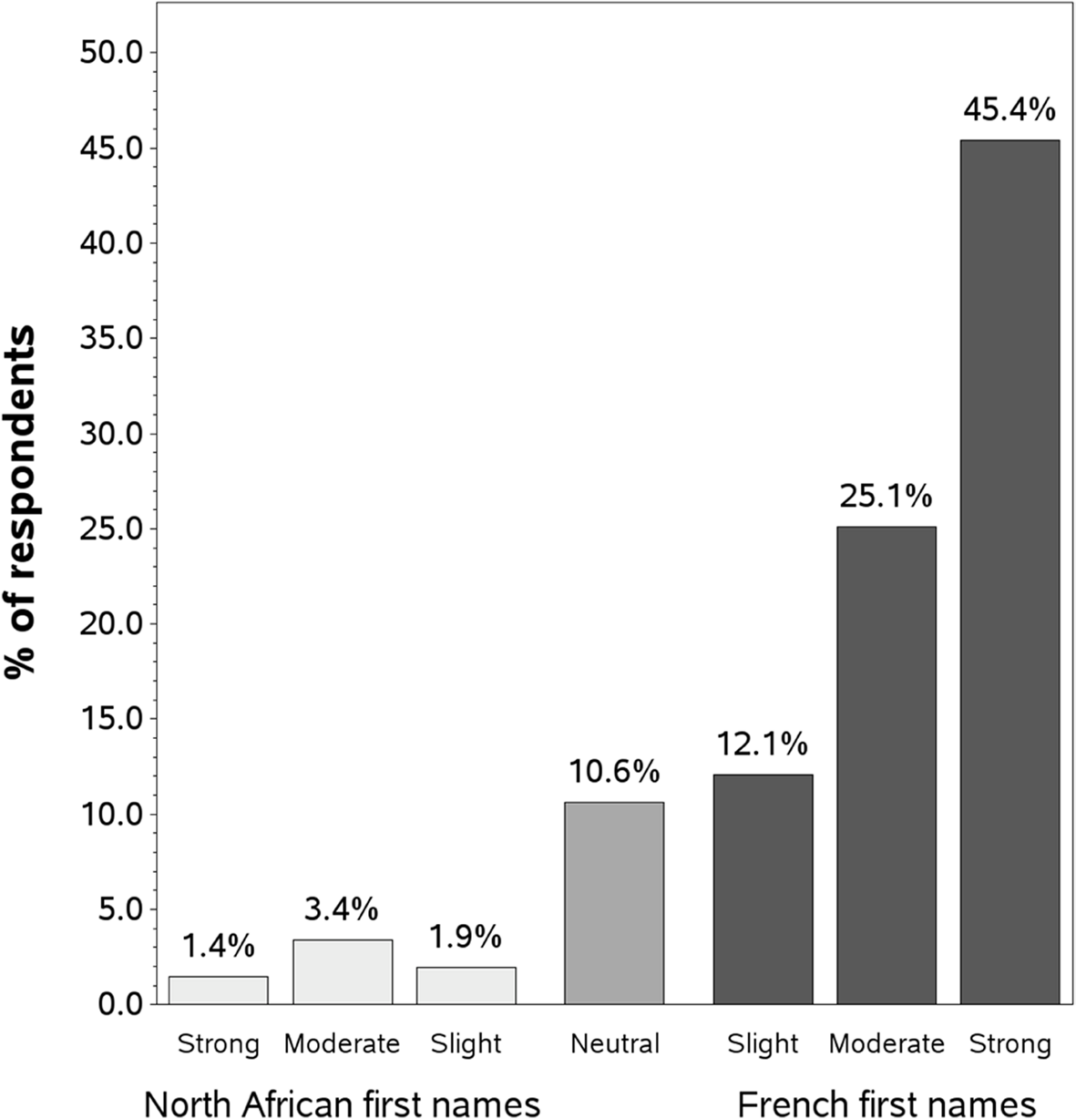
Distribution of implicit associations for first names associated with different ethnic groups

Figure 1 presents the distribution of implicit associations of positive/negative words with French-language first names compared with North African first names. The IAT mean score (D-score) was 0.53 (± 0.44), indicating a moderate association of positive words with French-language first names over North African first names. The majority of respondents (82.6%, CI 95%: 0.77 – 0.88) had a positive D-score, i.e.an implicit association of positive words with French-language first names. Several of the scores for implicit associations stand out.

Around 7% of the scores were negative, meaning that the trainees had an implicit association of positive words with North-African first names. Of the respondents with negative scores, 4.3% were Arab, 3.4% were White, and 1.9% were of mixed/other ethnic origin. The scores varied significantly according to ethnicity (χ2 = 88.6, *p* < 0.0001). In contrast, about 12% had a slight implicit association of French-language names with positive words, while 45.4% had a strong implicit association of French-language names with positive words.

It is also worth noting that approximately 10% of the respondents showed no group preference.

### Hudelson scale

Table 2 presents the perception of the relative responsibility to adapt to migrant patients in health care among trainee general practitioners. The five items of the Hudelson scale were moderately correlated (Pearson coefficients 0.09–0.46) and the internal consistency coefficient (Cronbach’s α) was 0.62. The average score for Hudelson scale was 18.9/35 (± 5.1), which means that adapting was considered to be mainly the responsibility of health care professionals. The majority of trainee GPs considered it to be mainly the health professionals’ responsibility to adapt when the patient expresses the wish to choose the sex of the health care professional, when he/she cannot read the language of the host country, and when his/her health beliefs contradict medical knowledge. Two items, however, differed from this trend. Roughly 38% of respondents considered it the responsibility of health care professionals to adapt to migrants when the patient does not speak the language of the host country. Approximately 35% considered it the responsibility of the patient to adapt and 27.5% considered it the responsibility of both the professional and the patient to adapt. This result is quite surprising because, when it comes to providing written information in the patient's language, respondents placed the responsibility on the health care professional (50.7%). When the migrant’s values and habits differ from those of the host country, however, GPs considered it to be the patient’s responsibility to adapt (58.5%).

**Table 2:** Relative responsibility to adapt of migrants and health professionals, as perceived by trainee GPs (2021): % per item (*n* = 207)

	**The responsibility lies with the health professionals**	**The responsibility lies with both health professionals and patients**	**The responsibility lies with the patients**	**Total**
When migrants' values and habits differ from those of the host country (%)	10.6	30.9	58.5	100
When the patient does not speak the language of the host country (%)	37.7	27.5	34.8	100
When the patient expresses the wish to be treated by a male or female doctor (%)	60.4	15.0	24.6	100
When the patient cannot read the language of the host country (%)	50.7	21.3	28.0	100
When the patient's health beliefs contradict medical knowledge (%)	46.4	26.1	27.5	100

There was a significant small positive correlation between implicit ethnic bias and cultural competence (*r* = 0.172, *p* < 0.05), suggesting that respondents for whom French-language names had more implicit positive associations were also more likely to consider it the migrants’ responsibility to adapt.

Table 3 provides the betas from the linear regressions for the factors associated with the IAT and with the Hudelson score. Respondents’ ethnic origins influenced the level of implicit ethnic bias. Being of Arab ethnicity or of other or mixed ethnic origin reduced the strength of implicit negative ethnic associations with North African first names (β = -0.54, *p* < 0.001, β =—0.29, *p* < 0.05 respectively). Having left-wing political opinions also had a negative correlation with the IAT score (β = -0.16, *p* < 0.05). Nevertheless, other individual and contextual variables such as age, sex, type of practice, practice area, frequency of contact with North Africans, and workload were not associated with the level of implicit bias.

**Table 3:** Factors associated with Implicit Association Test and with the Hudelson score: betas from the linear regression models

	**Implicit Association Test (-2, 2)**			**Hudelson score (5–35) **^**b**^		
**Covariates:**	β ^a^	CI_95%_	*p*-value	β	CI_95%_	*p*-value
**Age** (years)	0.00	(-0.03, 0.04)	0.93	0.03	(-0.09, 0.68)	0.13
**Sex** (ref = Male)						
Female	-0.05	(-0.18, 0.08)	0.49	**-2.02**	**( -3.46, -0.57)**	** < 0.01**
**Type of practice** (ref = Solo)						
Group practice	0.07	( -0.07, 0.21)	0.32	-1.24	( -2.75, 0.28)	0.11
Mixed practice	0.01	( -0.34, 0.37)	0.94	-2.68	( -6.61, 1.25)	0.18
**Ethnicity** (ref = White)						
Arab	**-0.54**	**( -0.76, -0.31)**	** < .0001**	-0.41	( -2.90, 2.08)	0.75
African	-0.08	( -0.40, 0.23)	0.60	1.57	( -1.96, 5.09)	0.38
Other or mixed	**-0.29**	**( -0.51, -0.07)**	**0.01**	-1.51	( -3.96, 0.94)	0.23
**Political opinion** (ref = Right-wing)						
Centre	0.03	(-0.17, 0.12)	0.72	-0.36	(-1.97,1.24)	0.66
Left (e.g. Socialist)	**-0.16**	**(-0.32, -0.01)**	**0.03**	**-3.07**	**(-4.74, -1.40)**	** < .001**
**Practice area** (ref = Urban)						
Suburban	-0.04	(-0.25, 0.16)	0.67	-1.92	(-4.30, 0.46)	0.11
Rural	-0.05	(-0.26, 0.17)	0.65	-0.42	(-2.94, 2.09)	0.74
**Frequency of contact with North Africans**	-0.05	(-0.10, 0.01)	0.07	0.13	(-0.49, 0.74)	0.69
**Workload** (no. of patients/day)	-0.00	(-0.01, 0.01)	0.95	0.06	(-0.06, 0.19)	0.30
**Proportion of patients of foreign origin**	-0.04	(-0.26, 0.17)	0.70	-0.37	(-3.00, 2.26)	0.78
**R**^**2**^**-range**	[0.16–0.24]			[0.09–0.17]		

^a^ β are adjusted for age, sex, ethnicity and type of practice; bold coefficients have *p*-value < 0.05

^b^ Low scores indicate that the responsibility to adapt is considered to lie with the health care professionals, whereas high scores indicate that it is considered to lie with the migrants

^c^ Table with all R-squared is in appendix 4

Concerning the Hudelson score, being a woman (β = -2.02, *p* = 0.01) was associated with considering it to be mainly the health professional’s responsibility to adapt to migrants. Respondents with left-wing political opinions also had a significant tendency to place the responsibility on health care professionals (β = -3.07, *p* < 0.01). Age, type of practice, having liberal political opinions, practice area, frequency of contact with North Africans, workload, and frequency of patients of foreign origin were not associated with considering it the health care professional’s responsibility to adapt to migrants.

The R^2^ ranged between [0.16–0.24] and [0.09–0.17] for the IAT score and the Hudelson score, respectively. Appendix 4 presents the details of the R^2^ for each factor.

### Reactions and feedback sharing

From the comments, it appears that some of the GPs were a bit sceptical about their implicit ethnic association scores and questioned the method and the existence of their own biases. A number of trainee GPs also highlighted the need for them to be aware of biases: “*Very impactful. It reveals that even unconscious biases can exist. This test confirms how important it is to know that these biases exist in order to be able to fight them consciously in our practice…*” (Participant 165).

We found few individual or contextual explanations in the regression analysis of implicit biases, aside from ethnicity. The influence of their ethnicity is also demonstrated in the comments: “o*ur culture played an unconscious bias in this test.*” (Participant 36).

Unlike the quantitative results, the association between political opinions and propensity to place the responsibility for adapting to migrants on health professionals was not addressed in the comments. Only one comment referred to political opinions: “*Surprised by the 60% [who said that the migrant should adapt to the values of the host country] … we are GPs not politicians… the main thing is the patient’s health.*” (Participant 132).

They also shared solutions with one another to implement in their practice as GPs. They suggested keeping the Hudelson scale in mind in their everyday practice. It will help them to remember that in most situations they bear responsibility for adapting to migrant patients. GPs also emphasized the importance of being conscious of one’s own biases in order to fight against them.

Finally, several trainees proposed “cultural openness” as a way to reduce the effects of these biases and improve the quality of care, such as working in an intercultural team, learning several languages and learning about different cultures.

## Discussion

### Main findings

This study set out to assess the trainee GPs’ implicit biases and their willingness to adapt care to diversity. Our results indicate that the majority of our trainees had a moderate to strong level of implicit bias favouring French-language first names over North African ones. This result applied to all sociodemographic characteristics, except ethnicity and political opinion. On the whole, trainees were willing to adapt care to migrants in most care situations, with two exceptions: when a patient did not speak the language of the host country and when the immigrants’ values and habits differed from those of the host country. This result applied to almost all sociodemographic groups.

### Implicit association test

The literature has often reported implicit associations with ingroups (and biases against exogroups) that are stronger than those found in this research [16]. Our study, however, does not support Allport’s finding that reduced implicit bias is associated with higher frequency of positive contact with the outgroup [23]. One possible explanation for this is the fact that we only assessed the frequency of contact with North Africans, not quality of that contact. Furthermore, the p-value for the variable “*frequency of contact with North Africans*” in the regression (Table 3) was 0.07, which suggests that either this measure needs to be assessed more accurately, for example, by including an assessment of the quality of trainee GPs’ relationships with members of the outgroup, or interaction with outgroup members does not reduce implicit bias among trainee GPs.

One important finding was that biases affect everyone and few protective factors exist in GPs’ working environments, apart from their own ethnicity and political opinions. This result has been demonstrated previously by Nosek, Banaji [24], who found a relationship between right-wing ideology and stronger implicit bias. They also concluded that bias could vary according to “*the constraints that culture imposes on individual attitudes*”, which could explain the influence of ethnicity on the IAT score. We found similar results in several studies, which showed that Black providers also had significantly weaker ethnic implicit associations for both White and Black people than White respondents [16, 25]. The authors, however, also found lower levels of ethnic bias among women, which was not the case with our results.

Our results suggest that completing an IAT and discussing the results could help to reduce the negative impact of bias on health professionals’ attitudes. Indeed, it could reduce both implicit and explicit bias among health providers with higher levels of implicit bias and thereby improve clinical interaction and verbal communication and make practice more patient-centred [26].

### Hudelson score

For three out of five situations, the majority of trainee GPs were willing to bear the responsibility to adapt care to migrants. They were more reluctant, however, to adapt to migrants’ values and habits. These findings are in line with those of Hudelson, Junod Perron [2]. In the respondents’ feedback, we noticed that some were surprised by this result. One of the respondents pointed out that they were GPs and not politicians. Our statistical analysis provides some explanations: adaptation is related to political orientation and the topic of values/habits is certainly a political issue, also from the GPs’ perspective. In a less competitive market, health care professionals may also feel less responsible for adapting to the values and habits of their patients as patients are free to move to another GP if they are not satisfied. One might thus speculate that the decrease in the percentage of GPs among all doctors in Belgium over the last decade has enhanced the market power of GPs [27].

Paradoxically, although more than half of the respondents see it as the health professional’s responsibility to adapt when the patient cannot *read* the language of the host country, opinion was spread evenly across the board when the patient cannot *speak* it. While Dauvrin et al. found that physicians took responsibility for providing interpreters [28], our respondents disagreed with that. One explanation could be the extra time and resources needed to provide an interpreter during an everyday consultation when GPs are often already under time pressure. Moreover, access to intercultural mediators remains limited in primary care.

A greater willingness to adapt was found among female GPs and those with left-wing political opinions. This is in line with previous research which found that female providers were more engaged in patient-centred communication and that African-American patients were more likely to be seen by female GPs [2]. Another explanation could also be that women tend to be more altruistic than men, when altruism requires more resources. We could therefore speculate that diversity might be considered as a form of altruism which demands more equalitarian behaviours, behaviours more likely to be found among women too [29].

The combination of the findings from the IAT and the Hudelson scale provides some support for the suggestion that there is a need to increase recognition and awareness of implicit ethnic bias and willingness to adapt to diversity in order to become more culturally competent. The weak correlation between the two concepts suggests that these are two different issues, requiring different interventions. The R^2^ was also weak for both the IAT scores and the Hudelson scores, meaning that our model did not fully explain the variation of these scores. Despite increased interest in providers’ biases in the literature and in medical education, there is still a lack of effective training methods. In a narrative review of the reduction of implicit bias in health care, Zestcott, Blair [30] suggested that public and professional awareness is a crucial starting point for efforts at reduction.

### Limitations

This study has some limitations, however, and the data must be interpreted with caution because they cannot be extrapolated to all trainee GPs. We recruited trainee GPs in their second year of internship from one important French-speaking university in Belgium, accounting for roughly a fifth of all trainee GPs in the country. In order to compare the data internationally, therefore, it would be useful for this study to be replicated in other Belgian medical schools and in other European countries. This study should be replicated among qualified GPs so that the results can be compared with those of trainees to see whether the extent of bias and willingness of trainee GPs to adapt to migrants changes over time. Further qualitative research is also required, such as in-depth interviews with respondents to gain a better understanding of their reactions.

We found several criticisms of the IAT regarding its construct validity and questioning the biases that it actually measures [31]. Finally, we considered it to be a reasonable research tool for assessing the level and distribution of implicit bias in a group [32]. Although the IAT has been criticized, most medical school programmes lack formal methods for assessing and reducing bias among medical students [33]. Moreover, completing an IAT also helps to reduce explicit biases via less direct mechanisms such as patient interaction and non-verbal behaviour [34].

## Conclusion

In conclusion, we believe that it is feasible to use the IAT to encourage trainees to engage with the topics of diversity and discrimination. The Hudelson scale was used to confront trainees with their levels of cultural competence. The debriefing on the results helped to raise awareness and sensitize the trainee GPs to the potential impact of their implicit and explicit biases in multicultural consultation. They also became aware that these biases could have an impact on their relationships and the way they care for patients.

One implication of this research is that it should be possible to use the results as a basis for reflection and discussion that could motivate health care professionals to tackle their ethnic biases. Another implication is the possibility of using the Hudelson scale as a tool to debate the extent of cultural competence among future GP. This debate could lead them to recognize and confront their own biases and to consider their responsibility as GP and how they could improve their intercultural interactions. The ethical approach to care, whereby a balance must be struck between patient autonomy, not doing harm, and helping could contribute to ensuring equitable healthcare in a diverse society [35]. According to Razai, Kankam [20], diversifying health care *“workforces improves the performance of the entire healthcare system”* and could improve the relationship between GPs and patients with different ethnic backgrounds.

Future research could be carried out among people with more diverse cultural backgrounds to compare the results at the level of implicit biases. We could also interview respondents after a year of practice, to see whether being aware and sensitized influenced their practice and reduced unintentional discrimination. Further research should be carried out to investigate the relationship between GPs’ implicit/explicit biases and attitudes towards migrant patients and the actual perceived discrimination reported by patients in Europe [4].

Another idea would be to give the IAT score and the Hudelson score to each respondent individually in order to investigate personal feedback in relation to the group’s average scores in more depth.

We hope that this research will provide a promising starting point for working towards a more ethnically equitable health care system.

## Supplementary Information

**Additional file 1: Appendix 1. **List of words used for the Implicit Association Test. **Appendix 2. **Hudelson scale. **Appendix 3.** Correlation between IAT and Hudelson scale. **Appendix 4. **R² for each covariate.

## Data Availability

The datasets used and/or analysed during the current study are available from the corresponding author on reasonable request. The datasets generated and analysed during this study are not publicly available due to the fact that consent was obtained from participants on condition that their data would not be shared, but are available from the corresponding author on reasonable request.

## Abbreviations

GP: General Practitioner
GDPR: General Data Protection Regulation
IAT: Implicit Association Test

## Footnotes

1 Cf. Myriatics. (2015, décembre). Myria. https://www.myria.be/fr/chiffres/myriatics

2 see Appendix 1.

3 see Appendix 2.
