# Supplementary material for: Implicit and explicit ethnic biases in multicultural primary care: the case of trainee general practitioners
Source: BMC Prim Care. 2022 Apr 21;23:91. doi: 10.1186/s12875-022-01698-8 (PMC9027448; doi:10.1186/s12875-022-01698-8)
Supplement: Supplementary file 1 — Additional file 1: Appendix 1. List of words used for the Implicit Association Test. Appendix 2. Hudelson scale. Appendix 3. Correlation between IAT and Hudelson scale. Appendix 4. R² for each covariate. [file 12875_2022_1698_MOESM1_ESM.docx]

# Appendix

## Appendix 1: list of words used for the Implicit Association Test

| Positive stimulus | Negative stimulus |
| --- | --- |
| Index 1: Love  Index 2: Happy  Index 3: Joy  Index 4: Beautiful  Index 5: Wonderful  Index 6: Peace  Index 7: Pleasure  Index 8: Laughter | Index 9: Ugly  Index 10: Injury  Index 11: Pain  Index 12: Failure  Index 13: Dreadful  Index 14: Horrible  Index 15: Evil  Index 16: Nasty |
| Stimulus French-language first names | Stimulus Moroccan first names |
| Index 17: Brigitte  Index 18: Caroline  Index 19: Julien  Index 20: Marie  Index 21: Nicolas  Index 22: Vincent | Index 23: Aziza  Index 24: Djamel  Index 25: Fatima  Index 26: Latifa  Index 27: Mohamed  Index 28: Rachid |

## Appendix 2: Hudelson scale

| 1. When immigrants' values and habits differ from those of the host country | | |
| --- | --- | --- |
| Host country institutions  should adapt to the  immigrants’ values and  habits | 1 2 3 4 5 6 7 | Migrants should adapt  to the values and habits  of the host country |
| 1. When the patient does not speak the language of the host   Country | | |
| The health professional  should always provide a  professional interpreter | 1 2 3 4 5 6 7 | It is the patient’s  responsibility to find an  interpreter |
| 1. When the patient expresses the wish to be treated by a male or female health professional | | |
| Hospitals should allow  patients who request it  to choose their health  professional’s sex | 1 2 3 4 5 6 7 | Patients should accept  being treated by the  health professional  provided by the  hospital, regardless of  their sex |
| 1. When the patient cannot read the language of the host country | | |
| Hospitals should  provide written  information in the  patient’s language | 1 2 3 4 5 6 7 | The patient should  arrange the translation of written information  provided by the hospital |
| 1. When the patient's health beliefs contradict the knowledge of the health professionals | | |
| The health professional  should adapt to the  patient’s beliefs  regarding the disease  and the treatment | 1 2 3 4 5 6 7 | The patient should trust  the explanations and  recommendations of the  health professional |

## Appendix 3: Correlation between IAT and Hudelson scale


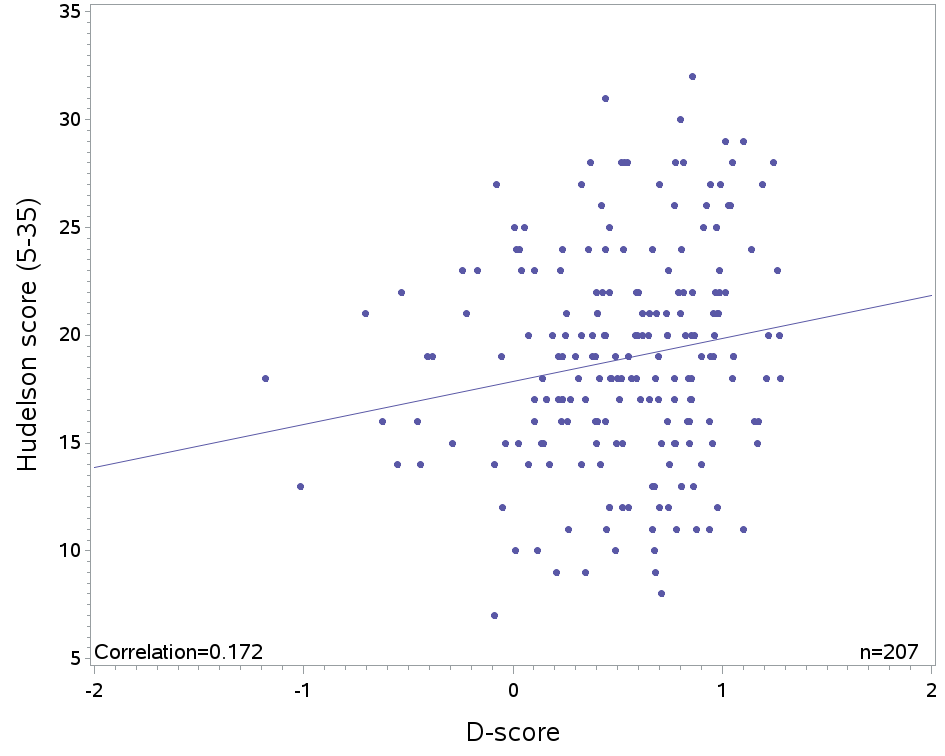


## Appendix 4: R² for each covariate

|  |  | IAT |  | |  | |  | | Hudelson | |  | |  |
| --- | --- | --- | --- | --- | --- | --- | --- | --- | --- | --- | --- | --- | --- |
| Covariates | RSquare | RootMSE | DepMean | |  | | RSquare | | RootMSE | | DepMean | |  |
| Political opinion | 0,16 | 0,40 | 0,54 | |  | | 0,17 | | 4,42 | | 18,54 | |  |
| Practice area | 0,24 | 0,41 | 0,51 | |  | | 0,11 | | 4,79 | | 18,89 | |  |
| Workload | 0,20 | 0,40 | 0,52 | |  | | 0,09 | | 4,84 | | 18,81 | |  |
| No. of contacts | 0,22 | 0,40 | 0,52 | |  | | 0,09 | | 4,83 | | 18,81 | |  |
| Proportion of contact with patient of foreign origin | 0,20 | 0,40 | | 0,52 | |  | | 0,08 | | 4,84 | | 18,81 | |
| Overall model (not included in average) | 0,18 | 0,40 | | 0,54 | |  | | 0,21 | | 4,34 | | 18,70 | |
